# A novel signal distribution-based approach to characterize atrial cardiomyopathy

**DOI:** 10.1007/s10840-026-02313-x

**Published:** 2026-04-13

**Authors:** Moritz T. Huttelmaier, Manuel Vogel, Jonas Herting, Jan Traub, Fabian Kerwagen, Luca F. Gerres, Anna Frey, Stefan Störk, Stefan Frantz, Caroline Morbach, Alexander Gabel, Thomas H. Fischer

**Affiliations:** 1 Department of Internal Medicine I, University Hospital Würzburg (UKW), Würzburg, Germany; 2 Comprehensive Heart Failure Centre Würzburg, University Hospital Würzburg, Würzburg, Germany; 3 Department of Infectious Diseases, Molecular Virology, Medical Faculty, Heidelberg University, Heidelberg, Germany

**Keywords:** Atrial fibrillation, Atrial cardiomyopathy, Multipolar electroanatomical mapping, Unsupervised machine learning

## Abstract

**Background:**

Modern endocardial contact-mapping displays myocardial substrate based on impaired signal amplitude (SA). Previous methods for characterizing left atrial (LA) myocardium rely on arbitrary thresholds and manual tracing of low-voltage areas. The amplitude and distribution of signals above these thresholds remain unexamined. This study aimed to develop novel parameters for characterizing LA myocardium and LA cardiomyopathy (AC) based on the analysis of the full distribution of SAs across the entire LA.

**Methods:**

We retrospectively identified 50 adult patients who underwent primary pulmonary vein isolation (PVI) for paroxysmal or persistent AF. Selection criteria were (i) electroanatomical mapping (EA) in sinus rhythm (SR) with ≥5000 points/map; (ii) transthoracic echocardiography (TTE) acquired in SR within 48 h prior to PVI. Exclusion criterion was previous LA ablation. The pulmonary veins were manually excised from the EA maps, and the SA of each point was plotted. A novel set of variables describing the voltage distribution across the entire LA was analyzed. Based on the assumption of healthier and sicker individuals within the study cohort, cluster analysis was performed for every voltage distribution variable using Gaussian mixture modelling. Associations with total LVA, NT-proBNP, CHA₂DS₂-VA score and LA TTE parameters were assessed (proof of function). AF recurrence rates following PVI were investigated.

**Results:**

The mean age of the studied sample (*n* = 48) was 62.5 ± 11 years, 62.5% were men, 66.7% showed persistent AF, median CHA_2_DS_2_-VA score was 1.5 (1, 3) and NT-proBNP was 190 (68, 413) pg/ml. A median of 5711 (5214, 6998) points/map were acquired. In this all-comer PVI cohort, mean SA was 2.4 ±0.96 mV, variance 2.27 ±1.07 mV^2^, skewness 1.43 ±0.59 and kurtosis 6.17 ±3.24. Variables did not differ between gender and subtypes of AF. For each variable, the sample was successfully stratified into groups mild and severe AC. Groups differed regarding NT-proBNP levels, CHA_2_DS_2_-VA score, LA TTE parameters and AF recurrence.

**Conclusion:**

We identified novel parameters to characterize LA myocardium by analyzing the full distribution of SAs across the entire LA using an open, investigator-independent strategy. SA-independent features including NTproBNP, CHA_2_DS_2_-VA score and echocardiographic measures of LA function consistently differed between mild and severe AC groups stratified by these novel parameters. This approach may not only facilitate a more precise characterization of AC but also advance automated analytic methods applicable during endocardial mapping and ablation of AF.

**Graphical abstract:**

**Figure:**
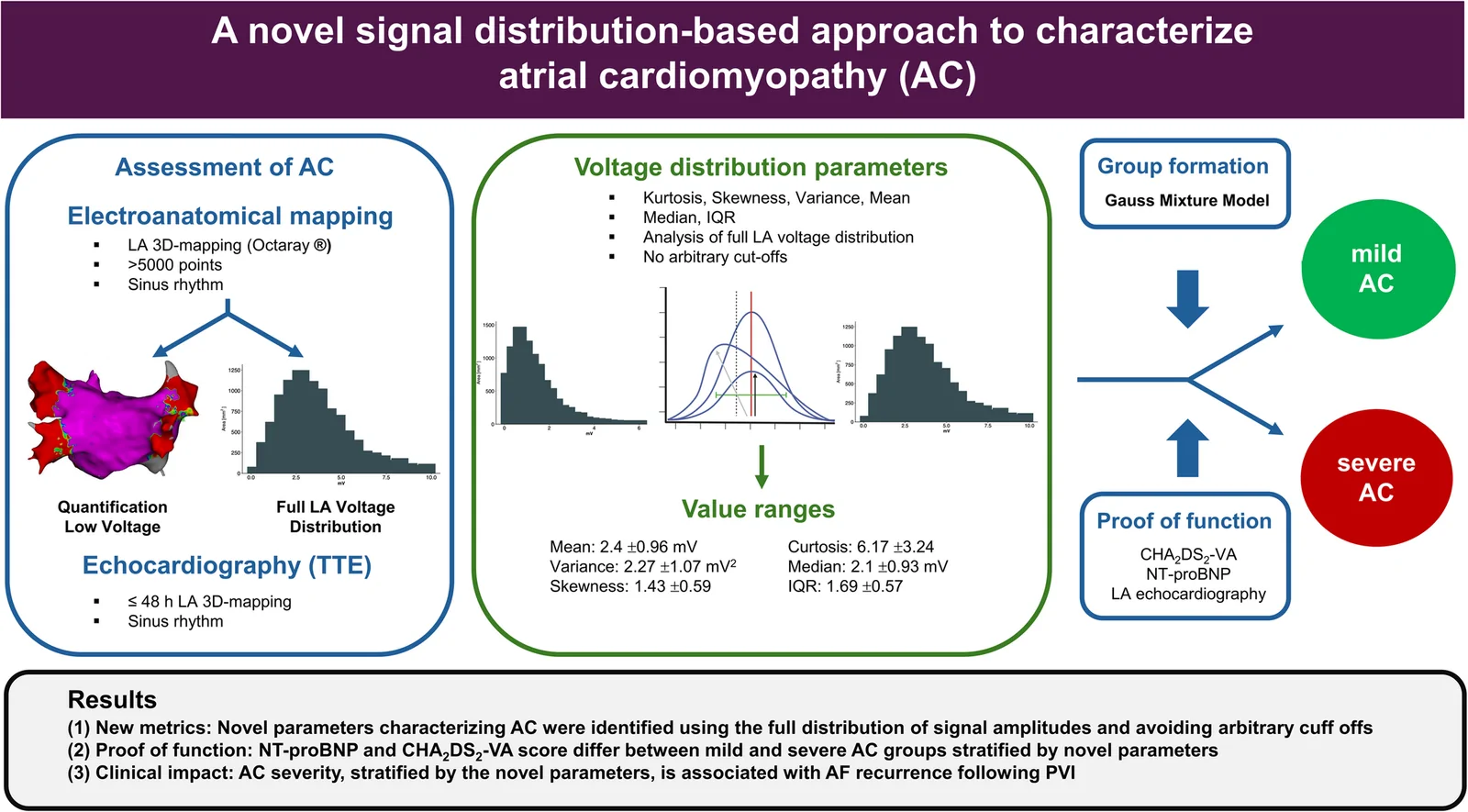
Amongst patients qualifying for pulmonary vein isolation, machine learning analysis of the full voltage distribution acquired during high-resolution endocardial contact mapping allowed to identify subgroups with mild and severe atrial cardiomyopathy avoiding the use of arbitrary cut offs. This approach may not only facilitate a more precise and individualized characterization of atrial cardiomyopathy but also advance automated analytic methods applicable during endocardial mapping and ablation of atrial fibrillation

**Supplementary information:**

The online version contains supplementary material available at https://doi.org/10.1007/s10840-026-02313-x.

## Introduction

Atrial fibrillation (AF) and atrial cardiomyopathy (AC) are pathophysiologically linked [1]. Establishing connections between left atrial (LA) mechanical dysfunction, electrical instability and structural remodelling, AC plays a central role in both the development and progression of AF [1, 2]. Diagnosis and severity of AC may impact AF treatment strategies [1]. Despite its clinical importance, noninvasive methods for the diagnosis of AC remain limited. By contrast, invasive endocardial contact-mapping offers a powerful tool for the characterization and diagnosis of AC through high-resolution site-specific signal amplitude analysis [3–8]. This method, however, is currently constrained through the reliance on (i) predefined, arbitrary voltage thresholds, (ii) manual delineation of low-voltage areas (LVAs) and (iii) the use of arbitrary LVA-cutoff values for the diagnosis of AC [3, 4, 9]. The amplitude and distribution of signals above these thresholds remain unexamined, individual and sex-specific variations are insufficiently characterized and there is no empirical validation for the LVA-cutoff values currently in use for the diagnosis of AC.

The aim of this study was to develop novel parameters for characterizing LA myocardium and LA AC based on the analysis of the full distribution of signal amplitudes across the entire LA using an open, investigator-independent strategy. This approach may not only facilitate a more precise characterization of AC but also advance automated analytic methods applicable during endocardial mapping and ablation of AF.

## Methods

**Fig. 1:**
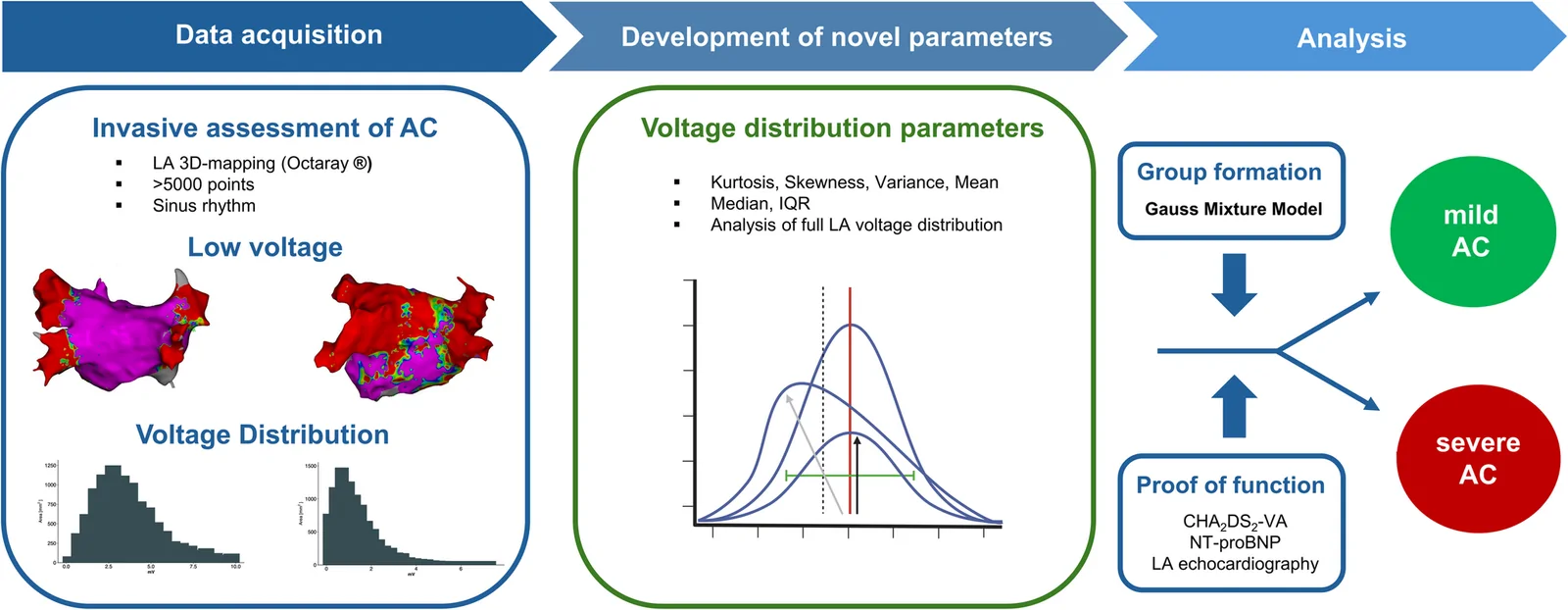
Illustration of methodological approach analyzing electroanatomical maps using an unsupervised machine learning approach

**Figure **1 summarizes and illustrates our methodological approach analysing electroanatomical maps with an unsupervised machine learning approach.

### Study sample

We retrospectively analyzed 50 adult patients who underwent primary pulmonary vein isolation (PVI) for paroxysmal or persistent AF between 08/22 and 05/23 at the University Hospital Würzburg. All consecutive patients who had undergone primary PVI using the CARTO→ electroanatomical mapping system during the study period were considered. Inclusion criteria comprised: (i) Electroanatomical mapping performed with a specific multipolar mapping catheter (OCTARAY™, Biosense Webster, Irvine, CA, USA); (ii) acquisition of voltage maps in sinus rhythm (SR) with ≥ 5000 points/map; (iii) transthoracic echocardiography (TTE) performed in SR within 48 h prior to PVI. Exclusion criteria included a history of LA catheter ablation or LA cardiac surgery. The study was conducted in accordance with the principles of the Declaration of Helsinki and its later amendments and approved by the Ethics Committee of the University Hospital Würzburg (reference number: 20230315 02). Baseline characteristics and key methodological details of the studied sample have been published previously [10].

### Clinical measurements and outcome ascertainment

#### Catheter ablation and electroanatomical mapping

All procedures were performed under deep analgesic sedation using fentanyl (25–100 micrograms) and propofol (400–700 mg/h). Local anesthesia for ultrasound-guided vascular access was achieved using mepivacaine. Airway support with an oropharyngeal (Guedel) airway was used routinely to maintain airway patency. All electro-anatomical maps were generated by two experienced interventional electrophysiologists with the same type of multipolar mapping catheter (OCTARAY™ 2-2-2-2-2, Biosense Webster, Irvine, CA, USA). For three-dimensional (3D) anatomical mapping the CARTO→ 3 system version 7 was used. We generated electroanatomical maps in SR with different upper voltage thresholds (0.2–0.5 mV and 0.2-1.0 mV). Wide circumferential PVI was performed using a contact force-enabled irrigated tip radiofrequency (RF) ablation catheter (Smart Touch Thermocool, tip electrode: 3.5 mm, Biosense Webster, Irvine, CA, USA) targeting for an ablation index (AI) of 350 at the posterior LA and an AI of 450 at the anterior LA and the carinas. Additional ablation lines were applied if LA flutter occurred during the procedure or if atypical LA flutter was previously documented and LVAs were present as a potential source of re-entry.

#### Echocardiography

Standardized transthoracic (TTE) echocardiography examinations (GE ultrasound system E95, probe 4Vc-D, GE Healthcare, Solingen, Germany) were performed by an experienced physician in accordance with current guidelines [11]. All off-line analyses were performed using the software ViewPoint 6 (ViewPoint 6 version 6.14, GE Healthcare, Solingen, Germany). We characterized left ventricular (LV) systolic and diastolic as well as right ventricular (RV) systolic function [11, 12]. Minimal and maximal two-dimensional (2D) LA volumes were measured in apical two- and four-chamber views, and minimal and maximal 3D LA volumes were quantified. Based on LA volumes we calculated 2D and 3D LA end-systolic volume index (LAVI), 2D and 3D LAVI/LV tissue Doppler relaxation velocity during atrial contraction (LAVI/a’), 2D and 3D total LA ejection fraction (EF), 2D and 3D active LAEF, and 3D passive LAEF. Longitudinal atrial strain representing LA reservoir, LA conduit and LA contraction were analysed semi-automatically off-line in two- and four-chamber views (2D) as well as based on a LA 3D dataset (3D) [13]. The zero-strain reference was set at the ventricular enddiastole (ed) and at the onset of atrial contraction (ac) in individual measurements.

#### Laboratory testing

Routine laboratory data obtained on the day preceding catheter ablation were retrieved from the institutional electronic medical record. In all patients, serum levels of N-terminal pro–B-type natriuretic peptide (NT-proBNP), creatinine, C-reactive protein (CRP), and glycated hemoglobin (HbA1c) were determined.

#### Operationalization of AC

We processed all LA electroanatomical maps uniformly prior to further analyses: The pulmonary veins (PVs) and the ventricular aspect of the mitral annulus were excluded. Processing and analyses of the electroanatomical maps were equally performed by a trained cardiac electrophysiologist certified by the European Heart Rhythm Association (EHRA). We applied two different methodological approaches (manual vs. semi-automated) for the operationalization of AC: For the *manual approach*, the LA electroanatomical map was divided into five anatomically defined sections (anterior, posterior, lateral wall, bottom and septum), LVAs were manually traced for each voltage threshold (0.2–0.5 mV; 0.2-1.0 mV) in every defined section and total LVA was calculated for every patient. For the s*emi-automated approach*, analyses were performed offline using the software CARTO-Net→. The signal amplitude (mV) of each point was plotted, and the distribution of signal amplitudes across 20 equal intervals, ranging from the minimum to the maximum signal amplitude, was assessed. A set of variables describing the voltage distribution across the entire LA (median, inter quartile range (IQR), mean, variance, skewness and kurtosis) was analyzed.

#### Outcome ascertainment

All patients underwent routine follow-up at our outpatient clinic three months after PVI, conducted by a board-certified cardiologist. The structured clinical evaluation included (i) physical examination, (ii) 12-lead surface ECG, (iii) a detailed medical history with the focus on symptomatic AF recurrence, and (iv) review of current pharmacotherapy. Ambulatory Holter ECG recordings obtained during outpatient visits were collected, analyzed, and stored in the institutional electronic medical record. At twelve to 20 months following the index PVI, all patients were recontacted for a structured telephone interview. In addition, the hospital’s documentation system was systematically reviewed for evidence of AF recurrences, and any available resting or Holter ECGs from external practices or external hospitals were retrieved and evaluated.

### Data analysis

Statistical analyses and machine learning approaches were implemented with the programming language R, version 4.4.2. (R Foundation for Statistical Computing, Vienna, Austria). We described data using mean (standard deviation, SD), median (IQR), or count (percent), as appropriate. Given the limited number of subjects and the assumption of healthier and sicker individuals within the study cohort, univariate cluster analysis was performed for every voltage distribution variable using a Gaussian mixture model (GMM), with 2 components (clusters). For parameter learning the expectation maximization (EM) algorithm was repeatedly started (100 runs). The maximum estimated cluster probabilities were used to assign each data point to a particular cluster. After fitting an independent GMM for each descriptive measure, normality within each resulting cluster was assessed visually using Q-Q plots and statistically by using the Shapiro-Wilk test (**Supplemental Fig. ****S1**). The cluster performance for each GMM based on a different voltage distribution parameter was calculated by the Silhouette score [14]. with the R package *cluster* [15]. We used total LVA as well as independent AC-markers (NT-proBNP, CHA₂DS₂-VA score, echocardiography) to verify the plausibility of correct group assignments. Furthermore, to highlight statistical differences of NT-proBNP, CHA₂DS₂-VA score, and echocardiographic parameter between the defined clusters, we performed a two-sided Mann-Whitney-U test. The Bonferroni-Yekutieli method was performed to correct for multiple testing, and adjusted p values below 0.05 were considered as statistically significant. The anonymised dataset as well as the detailed specification of the statistical analyses are available online (https://github.com/AlexGa/Distribution-based-approach-to-characterize-atrial-cardiomyopathy).

## Results

### Baseline characteristics

All consecutive patients (*n* = 60) who underwent primary PVI using the CARTO→ electroanatomical mapping system during the study period were considered. A total of *n* = 12 patients were excluded, predominantly because the TTE was not acquired in sinus rhythm prior to PVI. Mean age of the studied sample (*n* = 48) was 62.48 (± 11.01) years, 62.5% were men, and 66.7% showed persistent AF. The median CHA_2_DS_2_-VA score was 1.5 (quartiles 1, 3), and the median NT-proBNP level was 190 (68, 413) pg/ml. Diastolic dysfunction (grade 1–3) was present in 36.8% of study participants. In the studied sample, a median of 5711 (5214, 6998) points/map were acquired and the median LVA was 1.8 (0.5, 6.3) cm^2^ at the voltage threshold 0.2–0.5 mV and 7.8 (3.7, 24.6) cm^2^ at the voltage threshold 0.2-1.0 mV. The baseline characteristics of the total sample are further detailed in Table 1.

**Table 1:** Baseline characteristics of the total study sample

	Total sample *n*=48
Age (years)	64 (56-70)
Male sex, % (n)	62.5 (30)
Body mass index (kg/m²)	28 (24.9 - 30.9)
Atrial fibrillation	
CHA_2_DS_2_-VA score	1.5 (1-3)
Paroxysmal AF, % (*n*)	33.33 (16)
Persistent AF, % (*n*)	66.67 (32)
Antiarrhythmic medication, % (*n*)	39.58 (19)
Time from AF diagnosis to index PVI (days) *	716 (323 - 2114)
Cardiovascular risk factors	
Arterial hypertension, % (*n*)	77.1 (37)
Previous stroke or TIA, % (*n*)	4.2 (2)
Diabetes mellitus, % (*n*)	14.6 (7)
Electroanatomical mapping	
Mapping points per map, n	5711 (5213.5 - 6998)
Mapping time (min.) **	17 (12.5 – 19)
LVA cm^2^(<1.0 mV)	7.8 (3.7-24.6)
LVA cm^2^(<0.5 mV)	1.8 (0.5-6-3)
Laboratory values	
CRP (mg/dl)	0.1 (0.1-0.3)
eGFR (mg/ml/1.73 m^2^)	75 (63-85)
NT-proBNP (pg/ml)	190 (68-413)
HbA1c (%)	5.7 (5.5-5.9)
Echocardiography	
LVEF biplan (%)	61 (56-63)
GLPS 3D (-%)	17.6 (18.8 - 16.1)
E/e'	7.6 (6.1-9); *n*=47
TR Vmax (m/s)	2.5 (2.4-2.7); *n*=30
Outcome	
AF recurrence 12-20 months, % (n)	25 (12)

Linear trait values are presented as median with interquartile range (25th – 75th percentile). Relative and absolute frequencies of categorical variables are presented. *PVI* pulmonary vein isolation, *AF* atrial fibrillation, *LVA* low voltage area, *CRP* C-reactive protein, *eGFR* estimated glomerular filtration rate, *NT-proBNP* N-terminal pro-B-type natriuretic peptide, *LVEF* left ventricular ejection fraction, *GLPS* global longitudinal strain, *LAVI* left atrial volume index, *TR* Vmax=maximal velocity of tricuspid regurgitation.  *Time from AF diagnosis to index PVI was available for *n*=38 study participants, **mapping time was available for *n*=23 study participants

### Parameters characterizing LA voltage distribution

In LA AC, an altered voltage distribution – characterized by reduced signal amplitudes and increased heterogeneity – can be expected as a result of tissue remodelling and fibrosis. The following mathematical, parametric and non-parametric parameters were chosen for their potential to capture voltage magnitude and distribution variability: mean signal amplitude (meanSA), variance, skewness, kurtosis, median signal amplitude (medianSA) and IQR. In this all-comer PVI cohort, meanSA was 2.4 ± 0.96 mV, variance 2.27 ± 1.07 mV^2^, skewness 1.43 ± 0.59, kurtosis 6.17 ± 3.24, medianSA 2.1 ± 0.93 mV and IQR 1.69 ± 0.57. As expected, all parameters showed pronounced interindividual differences.

To evaluate potential individual and sex-specific differences in the voltage distribution parameters, subgroups were analyzed according to sex, age (young vs. old), AF subtype (paroxysmal vs. persistent), and comorbidities (diabetes, arterial hypertension (aHT), and prior stroke). **Supplemental Tables **S1 – S7 summarize the characteristics of these parameters across the different subgroups. No statistically significant differences were observed across subgroups defined by AF subtype or comorbidities. By contrast, sex-specific analysis revealed a consistent, non-significant trend toward higher meanSA, medianSA, and variance in men compared to women (meanSA: 2.8 vs. 1.9 mV; medianSA: 2.5 vs. 1.6 mV; variance: 2.9 vs. 1.3; all *p* = 0.07). Of note, analysis revealed significant age-related differences in parametric and non-parametric parameters, with younger participants showing higher meanSA, variance, medianSA, and IQR compared with older participants (meanSA: 3.8 vs. 1.9 mV, *p* < 0.001; variance: 7.9 vs. 1.4, *p* < 0.001; medianSA: 2.8 vs. 1.5 mV, *p* < 0.001; IQR: 2.5 vs. 1.4, *p* < 0.001).

### Characteristics in mild and severe AC stratified by LA voltage distribution parameters

**Fig. 2:**
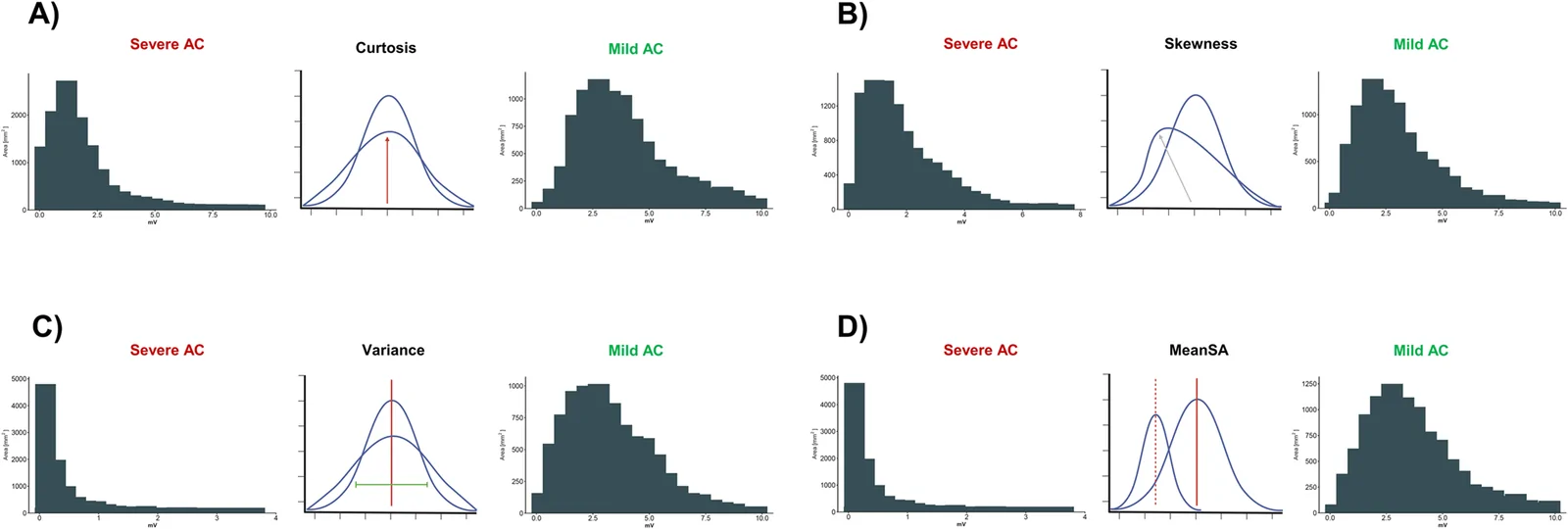
Graphical representation of voltage distribution parameters with exemplary curves from mild and severe AC groups and schematic illustration of each parameter. (**A**) Kurtosis. (**B**) Skewness. (**C**) Variance. (**D**) Mean signal amplitude (meanSA)

Given the assumption of healthier and sicker individuals within the study cohort, cluster analysis was performed with a GMM for every variable representing the voltage distribution. For each variable, patients were assigned to groups with a milder form of AC, further referred to as *mild AC* and those with a more severe form of AC, further referred to as *severe AC.* The clustering performance measured by the Silhouette score revealed highest values for kurtosis (0.74) followed by skewness (0.68), variance (0.65) and medianSA (0.60). In group *mild AC* kurtosis was 4.3 and 10.1 in group *severe AC* with higher values signifying a steeper peak of the voltage distribution, and lower values signifying flatter edges of the curve. Skewness was 1.1 in group *mild AC* and 2.0 in group *severe AC* with higher values of skewness indicating a more pronounced left-skewed distribution. Variance, characterizing the variation of voltages, was 2.84 mV^2^ in group *mild AC* and 0.92 mV^2^ in group *severe AC*. A higher variance indicates a greater dispersion of the data points from the mean, while a lower variance indicates less dispersion and a higher concentration of the data points around the mean. MeanSA was 3.1 mV in group *mild AC* and 1.6 mV in group *severe AC*. The non-parametric voltage distribution parameters, medianSA and IQR, were 2.7 mV and 1.9 in the *mild AC* group, and 1.2 mV and 1.5 in the *severe AC* group, respectively. Schematic illustrations of the different, parametric voltage distribution curves in the groups *mild AC* and *severe AC* are displayed in Fig. 2.

As further detailed in Table 2, the biomarker NT-proBNP, the clinical CHA₂DS₂-VA risk score and echocardiographic parameters characterizing LA form and function differed significantly between the groups *mild AC* and *severe AC* for every parameter investigated. Across all assessed parameters, there was a consistent, non-significant trend toward a higher percentage of persistent AF in the *severe AC* group.

**Table 2:** Patient characteristics in mild and severe AC stratified by LA voltage distribution parameters

	MeanSA			Variance			Kurtosis			Skewness		
	mild AC	severe AC	*p*	mild AC	severe AC	*p*	mild AC	severe AC	*p*	mild AC	severe AC	*p*
Value range	3.1 mV	1.6 mV		2.84 mV^2^	0.92 mV^2^		4.3	10.1		1.1	2.0	
NT-proBNP	134 (48-205)	425 (234-625)	<0.001	140 (56-230)	489 (286-722)	<0.001	139 (50-226)	472 (279-738)	<0.001	139 (50-226)	472 (279-738)	<0.001
CHA_2_DS_2_-Va	1 (0-1.2)	3 (2-4)	<0.0001	1 (0-2)	3 (2-4)	<0.001	1 (0.2-2)	2.5 (2-4)	<0.01	1 (0.2-2)	2.5 (2-4)	<0.01
Parox. AF % (n)	39.3 (11)	25 (5)	0.36	39.4 (13)	20 (3)	0.32	41.4 (13)	21.1 (3)	0.33	38.2 (13)	21.4 (3)	0.33
LAEF (3D)	42 (39-48)	39 (27-41)	0.05	41 (39-47)	33 (24-41)	0.05	41 (39-48)	36 (25-41)	<0.05	41 (39-48)	36 (25-41)	<0.05
LAVI/a‘	305 (222-365)	510 (344-895)	<0.05	333 (233-445)	631 (336-956)	<0.05	297 (231-365)	641 (452-955)	<0.001	297 (231-365)	641 (453-955)	<0.001
LA strain (ed, r)	24 (20-28)	15 (12-22)	<0.05	24 (18-28)	14 (12-19)	<0.05	24 (18-28)	14 (11-19)	<0.01	24 (18-28)	14 (11-19)	<0.01
Recurrence rates % (*n*)	28.6 (8/28)	60 (12/20)	0.04	30.3 (10/33)	66.7 (10/15)	0.03	29.4 (10/34)	71.4 (10/14)	0.01	29.4 (10/34)	71.4 (10/14)	0.01

	**median SA**						**IQR**					
	**mild AC**		**severe AC**		***p***		**mild AC**		**severe AC**		***p***	
	2.7		1.2				1.9		1.5			
NT-proBNP	139(48-191)		450(286-682)		<0.0001		140(50-210)		492(373-799)		<0.0001	
CHA_2_DS_2_-Va	1 (0-2)		3 (2-4)		<0.0001		1 (0.2-2)		2.5 (2-4)		<0.01	
Parox. AF (%, n)	41.4 (12)		21.1 (4)		0.21		38.2 (13)		27.3 (3)		0.33	
LAEF (3D)	43 (39-50)		38 (27-41)		<0.05		41 (39-47)		33 (24-40)		<0.05	
LAVI/a‘	317(237-400)		551(33-951)		<0.05		317(233-438)		631(368-956)		<0.01	
LA strain (ed, r)	24 (21-29)		14 (12-19)		<0.01		24 (18-29)		14 (11-19)		<0.01	
Recurrence rates % (*n*)	31 (9/29)		57.9 (11/19)		0.08		29.4 (10/34)		71.4 (10/14)		0.01	

Linear trait values are presented as median with interquartile range (25th – 75th percentile). Relative and absolute frequencies of categorical variables are presented. P-values refer to overall test across subgroups (Fisher’s exact test (dichotomous variables) or Mann-Whitney U-test (median)). *NT-proBNP* N-terminal pro-B-type natriuretic peptide (pg/ml), *AF* atrial fibrillation, *LAEF* left atrial ejection fraction, *3D* three dimensional, *LAVI/a’* left atrial volume index (LAVI)/LV tissue Doppler relaxation velocity during atrial contraction (a’),* ed* enddiastolic, *r *reservoir

Further, correlations between each parameter and signal amplitude-independent markers of AC (NT-proBNP, CHA₂DS₂-VA) were calculated. All examined parameters showed high correlations, with the parameter *meanSA* (-0.61 and − 0.64) exhibiting the strongest associations with NT-proBNP and CHA₂DS₂-VA, respectively. **Supplemental Fig.** S2–7 display the respective correlations for the parameters assessed.

### Association of LA voltage distribution parameters and LVA

**Fig. 3:**
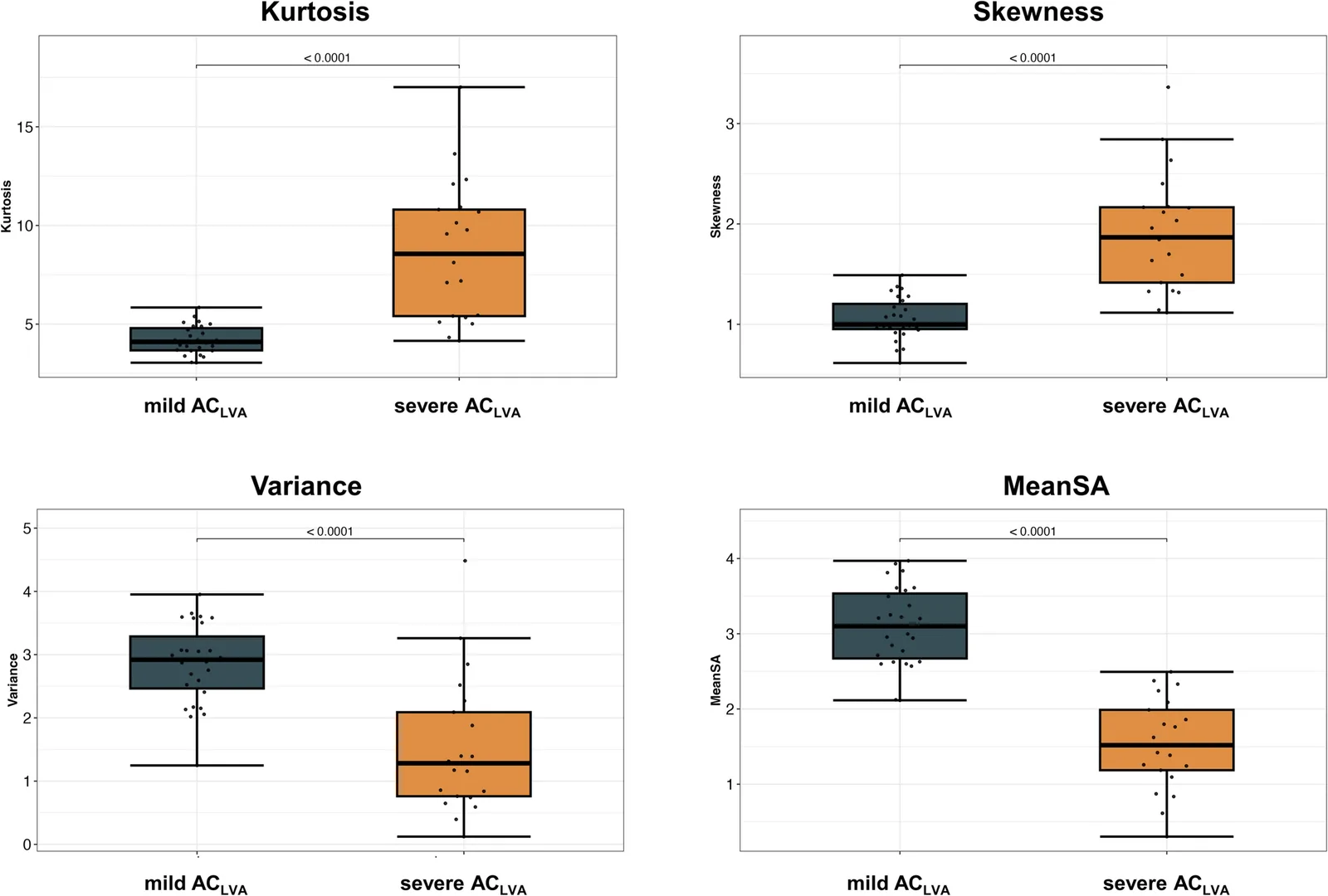
Distribution of voltage distribution parameter values across LVA-defined groups (*mild AC*_*LVA*_ *and severe AC*_*LVA*_). (**A**) Kurtosis. (**B**) Skewness. (**C**) Variance. (**D**) Mean signal amplitude

The parameters characterizing LA voltage distribution showed strong correlation with LA LVA: meanSA − 0.89, variance − 0.72, skewness 0.81, kurtosis 0.75, median − 0.87 and IQR − 0.84. The group assignments based on the LA voltage distribution parameters differed between previously defined and published groups *mild AC*_*LVA*_ and *severe AC*_*LVA*_ with LVA being used as the clustering variable [16]. Calculating the differences between these groups based on the novel parameters showed statistically significant differences (Fig. 3): kurtosis (*mild AC*_*LVA*_ 4.1 (3.7–4.8), *severe AC*_*LVA*_ 8.6 (5.4–10.8), *p* < 0.0001), skewness (*mild AC*_*LVA*_ 1.0 (1.0-1.2), *severe AC*_*LVA*_ 1.9 (1.4–2.2), *p* < 0.0001), variance (*mild AC*_*LVA*_ 2.9 (2.5–3.3), *severe AC*_*LVA*_ 1.3 (0.8–2.1), *p* < 0.0001), meanSA (*mild AC*_*LVA*_ 3.1, *severe AC*_*LVA*_: 1.5, *p* < 0.0001), medianSA (*mild AC*_*LVA*_ 2.8 (2.4–3.3), severe *AC*_*LVA*_ 1.3 (0.9–1.6), *p* < 0.0001), IQR (*mild AC*_*LVA*_ 2.0 (1.9–2.5), *severe AC*_*LVA*_ 1.3 (1.0-1.5), *p* < 0.0001).

### Association of LA voltage distribution parameters and AF recurrence

In the studied cohort, AF recurrence rates at up to 12 months were 25% and 41.7% at the follow up 12–20 months after index PVI. Structured telephone interviews and analyses of resting and Holter-ECGs revealed a significantly different rhythm stability between the groups *mild* and *severe AC*, stratified by the single parameters kurtosis, skewness, variance, mean and IQR. During the follow-up period (12–20 months), AF recurrence rates were consistently and significantly lower in group *mild AC* compared to group *severe AC*: kurtosis: 29.4% (*n* = 10/34) vs. 71.4% (*n* = 10/14) *p* = 0.01; skewness: 29.4% (*n* = 10/34) vs. 71.4%, (*n* = 10/14) *p* = 0.01; variance: 30.3% (*n* = 10/33) vs. 66.7% (*n* = 10/15) *p* = 0.03; mean SA: 28.6% (*n* = 8/28) vs. 60% (*n* = 12/20) *p* = 0.04; IQR: 29.4% (*n* = 10/34) vs. 71.4% (*n* = 10/14), *p* = 0.01.

## Discussion

The current study aimed to develop novel parameters for characterizing LA myocardium and LA AC based on the analysis of high-resolution electroanatomical maps in patients with paroxysmal or persistent AF. The following key findings emerged: (i) We identified novel parameters to characterize LA myocardium and LA AC by analyzing the full distribution of signal amplitudes across the entire LA using an open, machine-learning based strategy, thereby avoiding arbitrary cut-off values. (ii) Signal amplitude-independent features, including NT-proBNP, CHA_2_DS_2_-VA score, and echocardiographic measures of LA function, consistently differed between *mild* and *severe AC* groups assigned by these novel parameters. (iii) The novel parameters and the derived classification of AC were associated with the clinical outcome AF recurrence following PVI.

Modern endocardial contact-mapping offers a powerful tool for the characterization of LA myocardium through high-resolution site-specific signal amplitude analysis. This method, however, is currently constrained through the reliance on (i) predefined, arbitrary voltage thresholds, (ii) manual delineation of low-voltage areas (LVAs) and (iii) the use of arbitrary LVA-cutoff values for the diagnosis of AC: The majority of studies published to date that assessed the presence of AC using electroanatomical maps neither methodologically justify the bipolar LA voltage threshold value < 0.5 mV used to indicate low-voltage substrate nor the surface threshold values applied (> 5 cm^2^ respectively ≥2 cm^2^ respectively LVA ≥3% of LA surface) to define the presence of AC [3, 4, 9]. The amplitude and distribution of signals above these thresholds have so far been largely negotiated.

To our knowledge, this study uniquely investigates invasively assessed AC by analyzing the full distribution of signal amplitudes across the entire LA, thereby circumventing arbitrary cutoff values for AC classification and non-evidence-based thresholds for defining LA substrate. Moreover, the value ranges for the newly proposed parameters characterizing LA voltage distribution *meanSA*, *variance*, *skewness*, *kurtosis*, *medianSA* and *IQR* - are presented here for the first time, both for the overall cohort and for subgroups with *mild* versus *severe AC*.

### Voltage distribution parameters reflect structural and functional atrial remodeling in AC

The differing expression of LA voltage distribution parameters between the *mild* and *severe AC* groups is consistent with our pathophysiological rationale. Analysis of voltage distribution parameters revealed distinct patterns between patients with *mild* and *severe AC*. In *mild AC*, higher values of *meanSA*,* medianSA* and *variance* indicate generally preserved voltage amplitudes and a broader, more heterogeneous signal distribution, reflecting comparatively intact atrial myocardium. These findings are supported by echocardiographic measures of LA function: patients with *mild AC* exhibited significantly higher LAEF and LA strain, as well as lower LAVI/a’, collectively suggesting a more functional and healthier atrium. In contrast, patients with *severe AC* showed markedly lower *meanSA*,* medianSA* and *variance*, together with increased *skewness* and *kurtosis*, indicating a left-shifted, sharply peaked distribution (skewness) respectively heavier distribution tails (kurtosis) dominated by low-amplitude signals. These findings are consistent with progressive structural remodeling of LA tissue: As fibrosis and extracellular matrix deposition advance, electrically active myocardium is gradually replaced by non-conductive fibrotic tissue, leading to a reduction in overall voltage amplitude. The narrowing of the distribution and the heavier distribution tails (higher *kurtosis*) reflect a homogenization of the atrial substrate toward uniformly low voltages. Increased *skewness* further denotes a disproportionate accumulation of low-amplitude signals, consistent with areas of dense fibrosis. Conversely, the broader and flatter distribution in *mild AC* (lower *kurtosis*, higher *variance*) suggests a more heterogeneous LA myocardium, where healthy myocardium and early fibrotic regions coexist. These findings are supported by echocardiographic measures of LA function: patients with *severe AC* exhibited significantly lower LAEF and LA strain, as well as higher LAVI/a’, collectively suggesting a less functional and diseased atrium.

Altogether, these results support the concept that the full voltage amplitude distribution encodes important information about the extent and nature of atrial remodeling. The combination of decreasing *meanSA*,* medianSA* and *variance* with increasing *skewness* and *kurtosis* represents a characteristic electroanatomical signature of *severe AC* and may serve as a sensitive, quantitative marker of disease progression.

### Value ranges of voltage distribution parameters stratified by age, sex and subtype of AF

No statistically significant differences were observed across subgroups defined by AF subtype or comorbidities (diabetes, aHT, prior stroke). While sex-specific analysis revealed a consistent, non-significant trend toward higher values in men, age-related analysis demonstrated significant differences, with younger participants showing higher meanSA, variance, medianSA, and IQR values than older individuals. These results should be interpreted with caution given the limited sample size. From a pathophysiological standpoint, however, it appears plausible that men, who generally exhibit greater myocardial mass, and younger individuals, who are likely to have less advanced AC, demonstrate higher signal amplitudes [17, 18].

### Multipolar electroanatomical mapping technology

Recent advances in multipolar mapping catheters, such as the Octaray catheter (Biosense Webster, Irvine, CA, USA), enable the acquisition of electroanatomical maps with unprecedented spatial resolution and point density within increasingly shorter acquisition times. A strength of the present study lies in the high number of mapping points obtained for invasive electroanatomical assessment of the LA. The high spatial resolution is particularly crucial, as the LA myocardium is thin and early pathological processes such as inflammation or micro-ischemia may initially induce only subtle tissue alterations. To our knowledge, this is the first data set to achieve an average of more than 5711 (5214, 6998) mapping points per map during electroanatomical mapping in SR for the assessment of AC. The number of mapping points in previous studies assessing LA AC ranged from approximately 100 to 3000 per map [3–5, 9, 19, 20]. The substantially improved spatial resolution in the present dataset al.lows a detailed, fine-grained analysis of voltage amplitude distributions across the entire LA, (i) enabling the detection of subtle interindividual differences that may have remained unrecognized in earlier studies and (ii) form the basis for the novel methods of voltage distribution analysis introduced here.

### Rationale for unsupervised clustering to define mild and severe AC

Our approach, which assumed a continuous spectrum of AC severity within the studied cohort, deliberately avoided the use of predefined cutoff values for AC classification. Instead, unsupervised clustering was employed to identify subgroups based on intrinsic data structure, thereby eliminating the need for arbitrary voltage thresholds - a key conceptual advantage of our methodology. By focusing on the voltage distribution parameters, this approach addresses major methodological limitations of prior studies, such as the reliance on arbitrary cutoffs or merging LVA with other variables, early in the analysis. The pathophysiological validity of this clustering-based strategy is supported by the distinct differences observed between the mild and severe AC groups in clinical characteristics and outcomes previously associated with AC [3, 9, 21, 22]. Accordingly, the present dataset and analytical framework may provide a robust and reproducible foundation for invasive classification and characterization of AC.

### AF recurrence following PVI

Our data show a clear association of AC severity, assessed by invasive electroanatomical mapping, and AF recurrence rates twelve to 20 months after primary PVI. We are convinced that the association of AC severity and outcomes following AF ablation underlines not only the clinical significance of invasively assessed AC but also its potential impact on invasive AF treatment.

### Automatization of AC assessment

In previous studies using electroanatomical mapping for the classification and diagnosis of LA AC, LVAs were measured manually. This analytical step is not only time-consuming but also prone to measurement errors and considerable inter-operator variability. The parameters described in the present study, which evaluate the entire voltage spectrum after exclusion of the PVs, enable automated analysis and classification of AC, thereby overcoming these limitations. Although, for the sake of maximal precision, PVs were still manually delineated and excluded from the maps in the current study, this step can also be fully automated. In the near future, it may become feasible to display the severity of electroanatomically defined AC online during the creation of LA electroanatomical maps. The extent to which such real-time assessment should guide or support therapeutic decision-making during and beyond AF ablation will need to be determined in future prospective studies.

## Conclusion

We identified novel parameters to characterize the LA myocardium by analyzing the full distribution of signal amplitudes across the entire LA using an open, investigator-independent strategy. Signal amplitude-independent features including NTproBNP, CHA_2_DS_2_-VA score and echocardiographic measures of LA function, consistently differed between mild and severe AC groups stratified by these novel parameters. This approach may not only facilitate a more precise characterization of AC but also advance automated analytic methods applicable during endocardial mapping and ablation of AF.

### Limitations

No healthy participants without LA disease were included in this study. Although we examined a broad spectrum of patients, a bias of the measured parameters toward pathological values cannot be excluded, and normal reference values for a healthy LA cannot be defined based on the current data. Further studies including participants without LA disease are required to establish such reference values. Given the limited sample size, there remains a risk of overfitting despite the careful selection and validation of the applied machine learning methods. To enhance the generalizability of the model, future research should include (i) a larger and more heterogeneous patient population and (ii) external validation. Given the clinical objective of characterizing mild and severe AC, mixture models with two components were applied. However, due to the limited sample size, models with a higher number of components were not considered to avoid unstable parameter estimations and overfitting. It should be noted that the current analysis was performed exclusively using the CARTO mapping system with the Octaray catheter. As absolute voltage values may be influenced by catheter design, electrode size, spacing, and filtering characteristics, direct transfer of the parameter thresholds identified in this study to other mapping platforms will require dedicated system-specific validation. It must be further acknowledged that bipolar voltage amplitude is influenced by technical factors beyond myocardial pathology and that their potential contribution to the observed voltage distribution characteristics cannot be entirely excluded. Pulmonary vein exclusion was performed manually, introducing an element of operator dependency. Further, two patients required intraprocedural cardioversion prior to mapping. While AF duration in these cases was short (< 12 h), the potential influence of atrial stunning on mapping parameters cannot be entirely excluded. Finally, the number of resting and Holter-ECGs performed at twelve to 20 months following PVI varied between patients, increasing the susceptibility for error of our outcome analysis.

## Supplementary information

Below is the link to the electronic supplementary material.

Supplementary File 1 (DOCX 996 KB)

## Data Availability

The anonymised dataset as well as the detailed specification of the statistical analyses are available online (https://github.com/AlexGa/Distribution-based-approach-to-characterize-atrial-cardiomyopathy).
