# Supplementary material for: A novel signal distribution-based approach to characterize atrial cardiomyopathy
Source: J Interv Card Electrophysiol. 2026 Apr 13;69(5):977–87. doi: 10.1007/s10840-026-02313-x (PMC13585885; doi:10.1007/s10840-026-02313-x)
Supplement: Supplementary file 1 [file 10840_2026_2313_MOESM1_ESM.docx]

**Supplemental Material**

**-**

**A novel signal distribution-based approach to characterize atrial cardiomyopathy**

Moritz T. Huttelmaier, MD ^a^; Manuel Vogel, MD ^a^; Jonas Herting, MD ^a^; Jan Traub, MD ^a^; Fabian Kerwagen, MD MPH ^a,b^, Luca F. Gerres ^a^, Anna Frey, MD ^a,b^, Stefan Störk, MD PhD ^a,b^, Stefan Frantz, MD ^a,b^, Caroline Morbach, MD ^a,b^; Alexander Gabel, PhD ^c *^, Thomas H. Fischer, MD ^a *^

^a^ Dept. of Internal Medicine I, University Hospital Würzburg (UKW), Germany

^b^ Dept. Clinical Research & Epidemiology, Comprehensive Heart Failure Centre Würzburg, University Hospital Würzburg, Germany

^c^ Dept. of Infectious Diseases, Molecular Virology, Heidelberg University, Medical Faculty, Heidelberg, Germany

* these authors contributed equally

Corresponding address:

Fischer, T.H., MD

University of Wuerzburg – University Clinic

Department of Internal Medicine I

Oberdürrbacherstr. 6

D-97080 Würzburg, Germany

Phone: +49 931 201 39926

Email: Fischer_T@ukw.de

**Supplemental Table S1: Characteristics of LA voltage distribution parameters – gender**

|  | **Gender** | | |
| --- | --- | --- | --- |
|  | ***women*** | ***men*** | ***p*** |
| **meanSA** | 1.9 (1.2-2.6) | 2.8 (2.1-3.4) | 0.07 |
| **variance** | 1.3 (0.8-2.3) | 2.9 (2.2-3.1) | 0.07 |
| **skewness** | 1.3 (1.1-1.8) | 1.3 (1-1.5) | 0.5 |
| **kurtosis** | 5.1 (4.2-8.2) | 4.9 (3.8-5.7) | 0.5 |
| **medianSA** | 1.6 (0.9-2.3) | 2.5 (1.8-3.2) | 0.07 |
| **IQR** | 1.4 (1.3-1.8) | 2 (1.5-2) | 0.15 |

**Supplemental Table S2: Characteristics of LA voltage distribution parameters – age**

|  | **Gender** | | |
| --- | --- | --- | --- |
|  | ***young*** | ***old*** | ***p*** |
| **meanSA** | 3.8 (2.9-4.7) | 1.9 (1.2-2.6) | <0.001 |
| **variance** | 7.9 (4.6-14.6) | 1.4 (0.8-2.9) | <0.001 |
| **skewness** | 1.5 (1.2-2.1) | 1.8 (1.3-2.2) | 0.74 |
| **kurtosis** | 5.2 (3.4-8) | 7.6 (5.1-10.7) | 0.15 |
| **medianSA** | 2.8 (2.4-3.6) | 1.5 (0.9-2.3) | <0.001 |
| **IQR** | 2.5 (1.9-3.5) | 1.4 (1.1-2) | <0.01 |

**Supplemental Table S3: Characteristics of LA voltage distribution parameters – subtypes of AF**

|  | **Subtypes of AF** | | |
| --- | --- | --- | --- |
|  | ***Paroxysmal AF*** | ***Persistent AF*** | ***p*** |
| **meanSA** | 2.7 (2.1-3.2) | 2.4 (1.4-3.1) | 0.46 |
| **variance** | 2.6 (2-3.1) | 2.2 (1.2-3.1) | 0.53 |
| **skewness** | 1.2 (1-1.4) | 1.3 (1-1.9) | 0.66 |
| **kurtosis** | 4.6 (4.1-5.2) | 5.1 (3.9-8.8) | 0.65 |
| **medianSA** | 2.3 (2-2.8) | 1.9 (1.2-2.8) | 0.56 |
| **IQR** | 1.9 (1.5-2) | 1.5 (1.2-2) | 0.42 |

**Supplemental Table S4: Characteristics of LA voltage distribution parameters – diabetes**

|  | **diabetes** | | |
| --- | --- | --- | --- |
|  | ***No diagnosis of diabetes*** | ***Diagnosis of diabetes*** | ***p*** |
| **meanSA** | 2.6 (1.9 - 3.2) | 1.8 (1.4 - 2.3) | 0.23 |
| **variance** | 2.5 (1.4 - 3.1) | 1.3 (1 - 2.7) | 0.6 |
| **skewness** | 1.3 (1 - 1.7) | 1.3 (1.1 - 1.7) | 0.8 |
| **kurtosis** | 4.9 (3.9 - 7.2) | 5 (4.2 - 6.6) | 0.8 |
| **medianSA** | 2.4 (1.6 - 2.8) | 1.3 (0.9 - 2.2) | 0.23 |
| **IQR** | 1.9 (1.4 - 2) | 1.5 (1.3 - 1.7) | 0.48 |

**Supplemental Table S5: Characteristics of LA voltage distribution parameters – aHT**

|  | **aHT** | | |
| --- | --- | --- | --- |
|  | ***No diagnosis of aHT*** | ***Diagnosis of aHT*** | ***p*** |
| **meanSA** | 3.2 (2.7 - 3.4) | 2.2 (1.5 - 3) | 0.17 |
| **variance** | 2.9 (2.6 - 3.3) | 2.1 (1.2 - 3.1) | 0.29 |
| **skewness** | 1.1 (1 - 1.3) | 1.3 (1 - 1.9) | 0.52 |
| **kurtosis** | 4.4 (3.9 - 5) | 5.1 (4.2 - 8.6) | 0.39 |
| **medianSA** | 2.8 (2.3 - 3.1) | 1.9 (1.3 - 2.5) | 0.23 |
| **IQR** | 2 (1.7 - 2.2) | 1.5 (1.3 - 2) | 0.23 |

**Supplemental Table S6: Characteristics of LA voltage distribution parameters – presence of antiarrhythmic medication**

|  | **Presence of antiarrhythmic medication** | | |
| --- | --- | --- | --- |
|  | ***yes*** | ***no*** | ***p*** |
| **meanSA** | 2.4 (1.8 - 2.8) | 2.7 (1.6 - 3.2) | 0.6 |
| **variance** | 2.3 (1.4 - 3) | 2.5 (1.2 - 3.1) | 0.95 |
| **skewness** | 1.3 (1.1 - 1.6) | 1.2 (1 - 2) | 0.75 |
| **kurtosis** | 5 (4.2 - 6.5) | 4.9 (3.9 - 8.1) | 0.68 |
| **medianSA** | 1.9 (1.5 - 2.6) | 2.3 (1.3 - 2.8) | 0.68 |
| **IQR** | 1.5 (1.4 - 2) | 1.9 (1.4 - 2) | 0.61 |

**Supplemental Table S7: Characteristics of LA voltage distribution parameters – prior stroke**

|  | **Prior stroke** | | |
| --- | --- | --- | --- |
|  | ***no*** | ***yes*** | ***p*** |
| **meanSA** | 2.6 (1.8 - 3.2) | 1.4 (1.4 - 1.4) | 0.34 |
| **variance** | 2.5 (1.4 - 3.1) | 1 (0.9 - 1.1) | 0.31 |
| **skewness** | 1.3 (1 - 1.6) | 2 (1.8 - 2.2) | 0.33 |
| **kurtosis** | 4.9 (4 - 6.8) | 11 (10.3 - 11.6) | 0.23 |
| **medianSA** | 2.3 (1.5 - 2.8) | 1.4 (1.3 - 1.4) | 0.42 |
| **IQR** | 1.9 (1.4 - 2) | 1.2 (1.1 - 1.2) | 0.29 |

**Supplemental Figure S1:**

**Supplement Figure S1:** Validation of normality within clusters. Q-Q plots comparing theoretical Gaussian quantiles with empirical quantiles of voltage area distribution measures. P-values from Shapiro-Wilk tests shown for each cluster and measure. Left panels (A, C, E, G, I, K) represent mild AC clusters while the right panels (B, D, F, H, J, L) represent severe AC clusters. The majority of measures show no significant deviation from normality (p > 0.05), except IQR in mild AC (p < 0.05).

**Supplemental Figure S2:** Correlation of *meanSA* to NT-proBNP, CHA_2_DS_2_-Va and LVA

**Supplemental Figure S3:** Correlation of *variance* to NT-proBNP, CHA_2_DS_2_-Va and LVA

**Supplemental Figure S4:** Correlation of *curtosis* to NT-proBNP, CHA_2_DS_2_-Va and LVA

**Supplemental Figure S5:** Correlation of *skewness* to NT-proBNP, CHA_2_DS_2_-Va and LVA

**Supplemental Figure S6:** Correlation of *medianSA* to NT-proBNP, CHA_2_DS_2_-Va and LVA

**Supplemental Figure S7:** Correlation of *IQR* to NT-proBNP, CHA_2_DS_2_-Va and LVA

**Supplemental Figure S8:**

**Supplemental Figure S8:** Examples of electroanatomical maps acquired in sinus rhythm and the corresponding voltage distributions in patients with mild respective severe atrial cardiomyopathy.
